# Upfront haploidentical transplant for acquired severe aplastic anemia: registry-based comparison with matched related transplant

**DOI:** 10.1186/s13045-017-0398-y

**Published:** 2017-01-21

**Authors:** Lan-Ping Xu, Song Jin, Shun-Qing Wang, Ling-Hui Xia, Hai Bai, Su-Jun Gao, Qi-Fa Liu, Jian-Min Wang, Xin Wang, Ming Jiang, Xi Zhang, De-Pei Wu, Xiao-Jun Huang

**Affiliations:** 1 Peking University Institute of Hematology, Peking University People’s Hospital, No. 11 Xizhimen South Street, Xicheng District, Beijing, 100044 People’s Republic of China; 2 Beijing Key Laboratory of Hematopoietic Stem Cell Transplantation, Beijing, China; 3 Peking-Tsinghua Center for Life Sciences, Beijing, China; 4 The First Affiliated Hospital of Soochow University, Soochow, China; 5 Guangzhou First People’s Hospital, Guangzhou, China; 6 Xiehe Hospital affiliated to Huazhong University of Science and Technology, Wuhan, China; 7 Lanzhou Military Area General Hospital, Lanzhou, China; 8 The First Hospital of Jilin University, Changchun, China; 9 Nanfang Hospital Affiliated to Southern Medical University, Guangzhou, China; 10 Changhai Hospital affiliated to Second Military Medical University, Shanghai, China; 11 Shandong Provincial Hospital, Jinan, China; 12 The First Affiliated Hospital of Xinjiang Medical University, Urumchi, China; 13 Xinqiao Hospital Affiliated to Third Military Medical University, Chongqing, China

**Keywords:** Upfront, Haploidentical transplantation, Acquired severe aplastic anemia

## Abstract

**Background:**

Haploidentical donor (HID) hematopoietic stem cell transplantation (HSCT) is an alternative treatment method for severe aplastic anemia (SAA) patients lacking suitable identical donors and those who are refractory to immunosuppressive therapy (IST). The current study evaluated the feasibility of upfront haploidentical HSCT in SAA patients.

**Methods:**

We conducted a multicenter study based on a registry database. One hundred fifty-eight SAA patients who underwent upfront transplantation between June 2012 and September 2015 were enrolled.

**Results:**

Eighty-nine patients had haploidentical donors (HIDs), and 69 had matched related donors (MRDs) for HSCT. The median times for myeloid engraftment in the HID and MRD cohorts were 12 (range, 9–20) and 11 (range, 8–19) days, with a cumulative incidence of 97.8 and 97.1% (*P* = 0.528), respectively. HID recipients had an increased cumulative incidence of grades II–IV acute graft-versus-host disease (aGVHD) (30.3 vs. 1.5%, *P* < 0.001), grades III–IV aGVHD (10.1 vs. 1.5%, *P* = 0.026), and chronic GVHD (cGVHD) (30.6 vs. 4.4%, *P* < 0.001) at 1 year but similar extensive cGVHD (3.4 vs. 0%, *P* = 0.426). The three-year estimated overall survival (OS) rates were 86.1 and 91.3% (*P* = 0.358), while the three-year estimated failure-free survival (FFS) rates were 85.0 and 89.8% (*P* = 0.413) in the HID and MRD cohorts, respectively. In multivariate analysis, survival outcome for the entire population was significantly adversely associated with increased transfusions and poor performance status pre-SCT. We did not observe differences in primary engraftment and survival outcomes by donor type.

**Conclusions:**

Haploidentical SCT as upfront therapy was an effective and safe option for SAA patients, with favorable outcomes in experienced centers.

**Electronic supplementary material:**

The online version of this article (doi:10.1186/s13045-017-0398-y) contains supplementary material, which is available to authorized users.

## Background

Aplastic anemia (AA) is a rare but potentially fatal disorder characterized by hypocellular bone marrow and pancytopenia. Severe aplastic anemia (SAA) is a life-threatening type for which allogeneic hematopoietic stem cell transplantation (HSCT) and immunosuppressive therapy (IST) are the principle treatment modalities. Upfront matched related donor (MRD) HSCT is recommended for patients with SAA younger than 35 years of age [1] and, reportedly, results in long-term survival rates over 80% [2–5]. Unfortunately, only about 30% of patients have a matched sibling. According to current therapeutic algorithms, IST with a combination of horse antithymocyte globulin (ATG) and cyclosporin A (CsA) is the preferred first-line treatment for patients without a MRD, and HSCT from a haploidentical donor (HID) should be delayed until one or two courses of IST have failed [1]. However, approximately one third of patients did not respond to IST and one third of responders relapsed after initial therapy [5, 6]. In addition, clonal evolution was also observed in 15% of cases [7]. Furthermore, only rabbit and porcine ATG are available in China and are much less effective than horse ATG [8]. As such, IST has a high failure rate, which may impact patient quality of life due to the necessity for further treatment.

A study in the Asian Pacific observed that transplantation from alternative donors achieved comparable outcome to that from MRD, with a 5-year OS of 83.7 and 90.6% in pediatric patients [9]. Another study from European Cooperative Group for Bone Marrow Transplantation (EBMT) also demonstrated survival outcomes for upfront-unrelated donor HSCT similar to those of MRD HSCT in pediatric SAA (2-year OS of 96 and 91%) [10]. In recent years, remarkable improvement has also been made in HID SCT [11–13], primarily due to optimal conditioning regimens, improved supportive care, and advances in medications. However, published data on HID SCT for SAA are limited and mostly restricted in mixed variety line of therapy. In addition, the option of upfront HID SCT has never been investigated in a comparative manner. We previously developed a novel protocol and successfully used a salvage therapy for SAA patients [14]. To further assess the therapeutic effect and safety of upfront HID SCT for the treatment of SAA, we conducted a multicenter retrospective study based on data from a registry database.

## Methods

### Patients

Based on the Chinese Bone Marrow Transplantation Registry (CBMTR) registry database, 11 transplant centers in China that used the same protocol for HID HSCT in SAA patients were invited to join this study. Between June 2012 and September 2015, a total of 158 consecutive patients with acquired SAA underwent HSCT from a HID (*n* = 89) or MRD (*n* = 69) as upfront treatment. Disease severity was defined as previously described [15]. Patients with congenital bone marrow disorders (Fanconi anemia, Diamond-Blackfan anemia, and dyskeratosis congenital (DKC)) were excluded clinically and by laboratory assays. Trephine biopsy and chromosome tests were routinely performed. The chromosome breakage and gene test was used to exclude Fanconi anemia. Telomerase RNA component (TERC) mutation analysis was used to detect hidden forms of DKC, which was performed when congenital forms were suspected based on patient history and clinical analysis. The study was approved by the institutional review board of each of the eleven participating institutions, and written informed consent was obtained from all subjects in accordance with the principles of the Declaration of Helsinki.

### Conditioning regimen

Conditioning therapy in haploidentical transplantation consisted of the following: 0.8 mg/kg intravenous (i.v.) busulfan (BU) four times daily on days −7 and −6; 50 mg/kg i.v. cyclophosphamide (CY) once daily on days −5, −4, −3, and −2; and 2.5 mg/kg i.v. ATG (rabbit, Sangstat product Lyons, France) once daily for four consecutive days from days 5 to 2.

In matched related SCT, patients received either CY + ATG (CY: 50 mg/kg/day and ATG 2.5 mg/kg/day (rabbit)) or CY + ATG+ fludarabine (Flu) regimen. The three-drug conditioning consisted of 120–200 mg/m^2^ total Flu combined with 100–200 mg/kg total modified CY and 10–12.5 mg/kg total ATG.

### Stem cell mobilization and collection

The majority of patients received granulocyte colony-stimulating factor (G-CSF)-primed bone marrow (BM) combined with G-CSF-primed peripheral blood (PB) hematopoietic stem cell (HSC). Donor stem cell mobilization was performed using subcutaneous G-CSF (Filgrastim, Kirin, Japan or Granocyte, Chugai, Japan) at 5 μg/kg/day from day -3 until the last day of collection. BM grafts were harvested on day 1. The target mononuclear cell (MNC) count from BM was 2–4 × 10^8^/kg recipient weight. On day 2, peripheral blood stem cells (PBSCs) were collected by apheresis using a COBE Blood Cell Separator (Gambro BCT, Lakewood, CO, USA). The target MNC count from BM and PB was 6–8 × 10^8^/kg recipient weight. An additional harvest of PBSCs was performed on day 3 if the target MNC count was not achieved.

### GVHD prophylaxis, management, and supportive care

In the HID cohort, all patients received CsA, MMF, and short-term MTX as acute graft-versus-host disease (aGVHD) prophylaxis [16]. CsA (1.5 mg/kg, q 12 h, i.v.) was administered from day −9 with a target trough concentration of 150–250 ng/mL. It was switched to oral administration when recipient bowel function returned to normal. From day −9, MMF (0.5 g q 12 h, 0.25 g q 12 h in pediatric patients) was administered orally, which was tapered to half on day +30 and discontinued on day +60. MTX (15 mg/m^2^) was administered intravenously on day +1, followed by a dose of 10 mg/m^2^ on days +3, +6, and +11 (in MRD HSCT, MTX +1, +3, and +6). In the MRD cohort, MMF dosage was tapered upon engraftment.

Acute and chronic GVHD (aGVHD and cGVHD) were diagnosed and graded according to international criteria [17, 18]. With regards to the treatment of aGVHD, the effective concentration of CsA was resumed, and 1–2 mg/kg/day methylprednisolone equivalents were administered as first-line therapy. For steroid-refractory aGVHD, second-line treatments such as tacrolimus (FK506), MMF, and CD25 monoclonal antibody (CD25 mAb) or MTX were administered.

G-CSF (5 μg/kg/day) was administered subcutaneously from day +6 until myeloid engraftment in the HID but not in the MRD cohort. Viral surveillance included screening for cytomegalovirus (CMV) and Epstein-Barr virus (EBV) by polymerase chain reaction twice weekly. Preemptive ganciclovir (DHPG) or foscarnet therapy was initiated for positive CMV polymerase chain reaction findings. Other infection prevention and supportive care were provided in accordance with previous articles [19, 20].

### Definitions and evaluation

Myeloid and platelet engraftment were defined as previously described [21]. Full donor chimerism (FDC) was defined as the presence of >95% donor hematopoietic cells. Patients who did not reach neutrophil counts >0.5 × 10^9^/L for three consecutive days after transplantation were considered to have had primary graft failure. Patients with initial engraftment in whom recurrent pancytopenia with obviously hypocellular BM and without moderate to severe acute GVHD were considered to have had secondary graft failure [22]. OS was defined as the time from the date of HSCT to death from any cause or last follow-up. Failure-free survival (FFS) was defined as survival with response. Death, primary or secondary graft failure, and relapse were considered treatment failures. GFFS (GVHD-free, failure-free survival) was defined as grades III–IV acute GVHD, extensive chronic GVHD, and treatment failures as the above. Transplantation-related mortality (TRM) was defined as death without relapse. Regimen-related toxicity was evaluated according to Seattle Toxicity Criteria [23]. Performance status was graded according to Eastern Cooperative Oncology Group (ECOG) scoring.

### Statistical analysis

The last follow-up for all surviving patients was April 30, 2016. Patients lost to follow-up were censored at the time of their withdrawal. Differences in the distribution of various parameters for the two groups were compared using chi-square or Student’s *t* tests as appropriate. Analyses of OS, FFS, and GFFS were performed using the Kaplan–Meier method, with differences compared by log-rank tests. Cumulative incidences of engraftment and GVHD were estimated in the competing risk model, with early death as the competing event. Univariate and multivariate analyses were performed to determine whether any of the selected factors were predictive of the endpoints. In multivariate analysis, all factors with *P* < 0.1 in univariate analysis were evaluated in the Cox regression model with a backward stepwise model selection approach. Significant factors (*P* < 0.05) were considered to be independently predictive of the outcomes. All statistical analyses were performed using SPSS Version 13.0 and R software package (version 2.6.1; http://www.r-project.org).

## Results

### Basic characteristics

Table 1 shows the patient and transplant characteristics of the 158 patients. All patients had no IST or had only CsA as IST for a limited time before transplantation. All cases underwent transplantation within 4 months after definite diagnosis. The two cohorts were similar with regard to male/female ratio, interval between diagnosis and transplant, red blood cell (RBC) transfusions, and ECOG scores prior to transplant. HID patients were younger than patients in the MRD cohort (median age 22 years [range, 4–51 years] vs. 33 years [range, 7–61 years], respectively; *P* < 0.001). Recipients of mismatched transplants had more male donors than did the recipients of matched transplants. Approximately 90% of patients in the HID group received grafts from BM and PB, compared to 63.2% in the MRD cohort. The graft compositions in the two groups were similar except that the matched patients had higher CD34 cell counts (4.5 [range, 1.5–30.2] × 10^6^/kg vs. 3.6 [range, 0.5–18.8] × 10^6^/kg, respectively; *P* = 0.001).

**Table 1:** Patient and graft characteristics

Variable	Haploidentical *N* = 89	Matched related *N* = 69	*P*
Age (years), median (range)	22 (4–51)	33 (7–61)	<0.001
Children, no. (%)	33 (37.1%)	7 (10.1%)	<0.001
Adult, no. (%)	56 (62.9%)	62 (89.9%)	
Male/female, no.	57/32	39/30	0.337
SAA/VSAA, no.	69/20	58/11	0.305
Disease course (months), median (range)			0.507
Interval from SAA diagnosis to SCT	1.5 (1–4.0)	1.0 (1–3.0)	
Previous transfusion			
Median units of RBC (range)	7.0 (0–30)	8.0 (2–34)	0.115
Median units of PLT (range)	13.5 (0–90)	10.0 (2–90)	0.036
^a^ECOG pre-SCT, median, (range)	1 (0–3)	1 (0–2)	0.568
Donor-patient sex match, no. (%)			0.042
Male-male	40 (44.9%)	17 (24.6%)	
Male-female	19 (21.3%)	15 (21.7%)	
Female-male	19 (21.3%)	22 (31.9%)	
Female-female	11 (12.4%)	15 (21.7%)	
Donor type, no. (%)			<0.001
Sibling	25 (28.1%)	69 (100%)	
Parent	57 (64.0%)	–	
Child	5 (5.6%)	–	
^b^Others	2 (2.2%)	–	
HLA type, no (%)			<0.001
6/6	4 (4.5%)	69 (100%)	
5/6	6 (6.7%)	–	
4/6	21 (23.6%)	–	
3/6	58 (65.2%)	–	
ABO matched, no. (%)			0.414
Matched	49 (55.1%)	44 (63.8%)	
Minor mismatched	18 (20.2%)	13 (18.8%)	
Major mismatched	14 (15.7%)	10 (14.5%)	
Different	8 (9.0%)	2 (2.9%)	
Graft type, no. (%)			<0.001
BM + PB	78 (87.6%)	43 (63.2%)	
BM only	9 (10.1%)	3 (4.4%)	
PB only	2 (2.2%)	22 (32.4%)	
Median MNCs, ×10^8/kg (range)	9.9 (3.4–32.0)	10.5 (4.6–26.4)	0.817
Median CD34+ count, ×10^6/kg (range)	3.6 (0.5–18.8)	4.5 (1.5–30.2)	0.001
Median CD3+ count, ×10^8/kg (range)	1.8 (0.1–6.7)	2.1 (0.1–7.7)	0.530
Median CD4+ count, ×10^8/kg (range)	1.0 (0.1–3.5)	1.1 (0.1–5.0)	0.337
Median CD8+ count, ×10^8/kg (range)	0.7 (0.1–3.0)	0.8 (0.1–2.7)	0.147
Median follow-up among alive patients, mo. (range)	21.4 (7.1–47.6)	26.0 (7.5–47.6)	0.258
Neutrophil engraftment, median (range)	12 (9–20)	11 (8–19)	0.151
Platelet engraftment, median (range)	15 (6–91)	14 (7–36)	0.484

*BM* bone marrow, *PB* peripheral blood, *MNC* mononuclear cell

*Patient age, previous transfusion of platelet, donor-recipient sex match, graft type, and infused CD34 cells differed significantly between the two groups (*P* < 0.05). There were no other significances between group differences

^a^ECOG (Eastern Cooperative Oncology Group scale) is used to evaluate patients’ performance status

^b^Other donor types were from cousins

### Engraftment

In the HID group, 87 of 88 (98.9%) cases who survived more than 28 days achieved myeloid engraftment at a median of 12 days (range, 9–20 days). In the MRD cohort, 67 evaluable patients engrafted at a median of 11 days (range, 8–19 days). The cumulative incidences of 28-day engraftment were 97.75 ± 0.03% and 97.10 ± 0.05% (*P* = 0.528, Additional file 1: Figure S1a) in the mismatched and matched groups, respectively. Eighty-six and 66 patients had platelet engraftments in the HID and MRD groups at 15 days (range, 6–91 days) vs. 14 days (range, 7–36 days), with cumulative incidences of platelet engraftment of 96.63 ± 0.05% and 95.65 ± 0.08%, respectively (*P* = 0.989, Additional file 1: Figure S1b). One HID patient failed to achieve primary engraftment and underwent a second transplant from the original donor; however, he experienced primary graft failure again. One patient in the MRD cohort experienced secondary graft failure on day +45 and refused further therapy.

### GVHD

The cumulative incidence of grades II–IV aGVHD at 100 days were 30.34 ± 0.24% and 1.45 ± 0.02% after HID and MRD transplants, respectively (*P* < 0.001, Additional file 1: Figure S2a). The cumulative incidence of grades III–IV aGVHD at 100 days were 10.11 ± 0.10% and 1.45 ± 0.02% (*P* = 0.026, Additional file 1: Figure S2b). Multivariate analysis identified no significant factors in II–IV aGVHD, and HID was the only independent factor associated with III–IV aGVHD (Table 2).

**Table 2:** Multivariate analysis of adverse factors associated with survival outcomes and GVHD

Outcome	Hazard ratio (95% confidence interval)	*P* value
Overall survival		
Previous RBC (>10)	6.8 (1.9–23.8)	0.003
ECOG (>2)	2.9 (1.1–7.9)	0.032
Failure-free survival		
Previous RBC (>10)	5.4 (1.8–16.3)	0.003
ECOG (>2)	3.0 (1.2–7.5)	0.022
II–IV aGVHD		
HID	1.3 (0.9–1.8)	0.181
III–IV aGVHD		
HID	1.6 (1.2–2.2)	0.006

*HID* haploidentical donor, *aGVHD* acute graft-versus-host disease, *cGVHD* chronic graft-versus-host disease

Eighty-three and 65 patients in the HID and MRD cohorts, respectively, with survival longer than 100 days after transplantation were evaluable for the incidence of cGVHD. HID patients had a higher three-year cumulative incidence of cGVHD than did the MRD patients (39.30 ± 0.54% vs. 8.35 ± 0.13%, *P* < 0.001, Additional file 1: Figure S3a). However, the two groups had similar three-year incidences of extensive cGVHD (3.42 ± 0.04% vs. 2.03 ± 0.04%, *P* = 0.426, Additional file 1: Figure S3b). During the follow-up, three mismatched and one matched patients with extensive cGVHD received systemic therapy.

### Infectious complications and immune reconstitution

The most common infection was the reactivation of CMV, which occurred in 46 (51.7%) HID and 30 (43.5%) MRD patients (*P* = 0.306), at a median of 30 (range, 16-74) and 28 (range, 11-49) days post-transplantation. Only one HID patient developed CMV enteritis on day +33 and recovered after administration of antiviral drugs combined with an infusion of CMV-specific cytotoxic T lymphocytes (CMV-CTL). Twenty-five (28.1%) and 15 (21.7%) suffered EBV viremia in the HID and MRD-SCT groups (*P* = 0.363). The median times to EBV viremia in the two cohorts were 41 (range, 26–73) and 34 (range, 18–89) days, respectively. One HID and one MRD case developed EBV-associated post-transplant lymphoproliferative disorders (PTLD) on days +76 and +68, respectively.

**Fig. 1:**
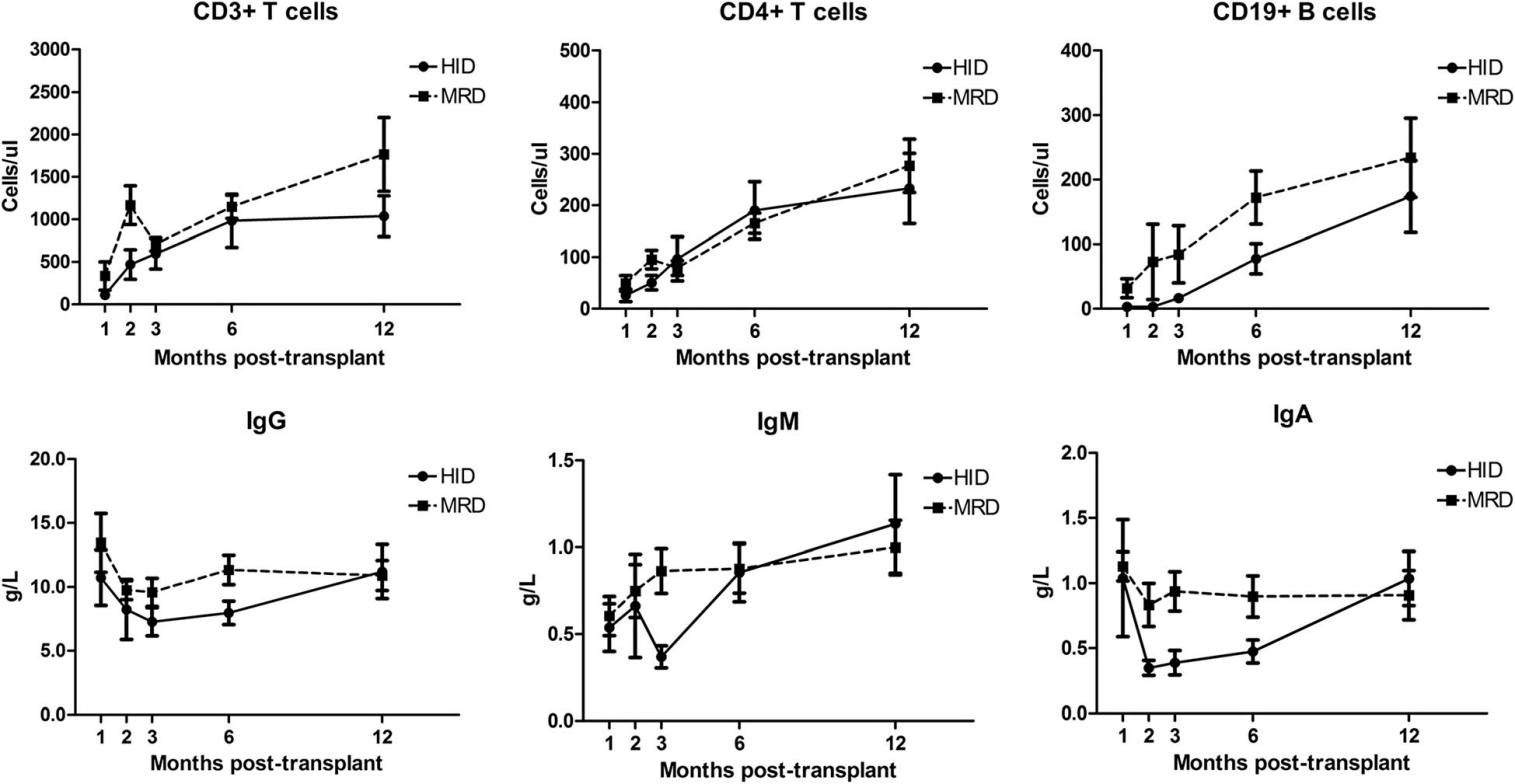
Immune reconstitution. Reconstitution of CD3, CD4, and CD19 lymphocytes were comparable from 6 months post-SCT. Equivalent levels of immunoglobulins A, G, and M (IgA, IgG, IgM) were achieved at 1 year between two cohorts

The outcomes of immune reconstitution are shown in Fig. 1. CD3, CD4, and CD19 concentrations were comparable between the two cohorts from 6 months post-SCT. Furthermore, equivalent levels of immunoglobulins A, G, and M (IgA, IgG, IgM) were achieved at 1 year.

### Transplantation-related mortality

During a median follow-up of 22.6 months (range, 7.1–47.6), 12 and 6 were in the HID and the MRD groups, respectively, with a median time to death of 96.5 (range, 2–345 days) and 51 days (2–244 days). Analyses of TRM revealed that GVHD and infection were the major causes of death in the two groups. In the HID cohort, six patients (6.74%) died of infection (two fungal, one EBV-associated PTLD, and three serious bacterial infections), four (4.49%) died of GVHD (three severe aGVHD and one extensive cGVHD), one (1.12%) of regimen-related toxicity (RRT), and one of primary graft failure. Six (8.70%) patients died of TRM in the MRD cohort, which included three (4.35%) of infection (two fungal and one bacterial), one (1.45%) due to severe aGVHD, one from RRT, and one from secondary graft failure.

### Survival outcomes and follow-up

**Fig. 2:**
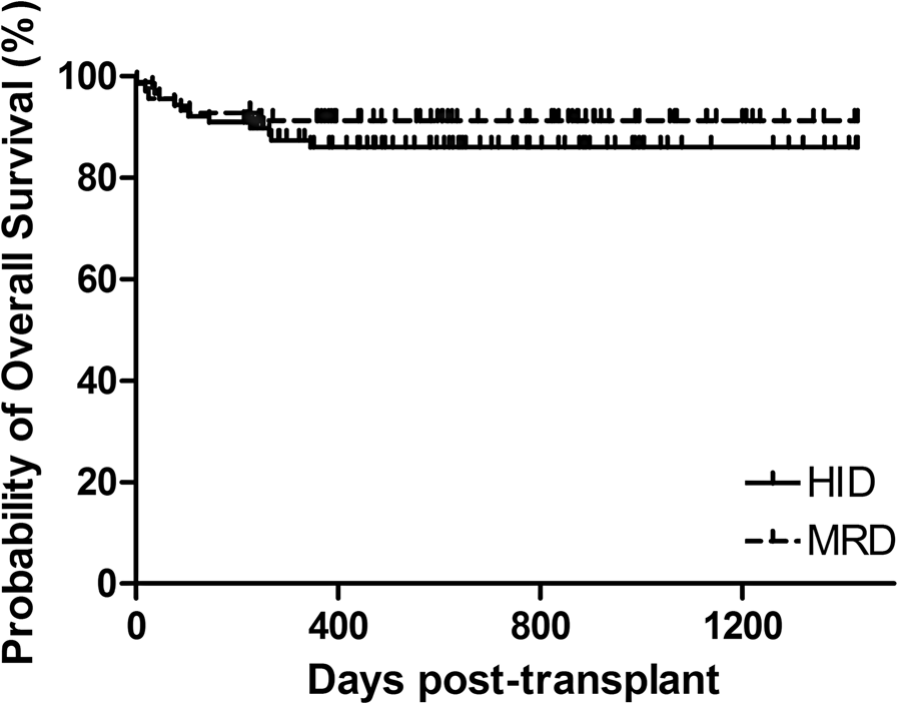
Overall survival of two cohorts: HID, 3-year OS of 86.1% ± 3.7%; MRD, 3-year OS of 91.3% ±3.4% (*P* = 0.358)

**Fig. 3:**
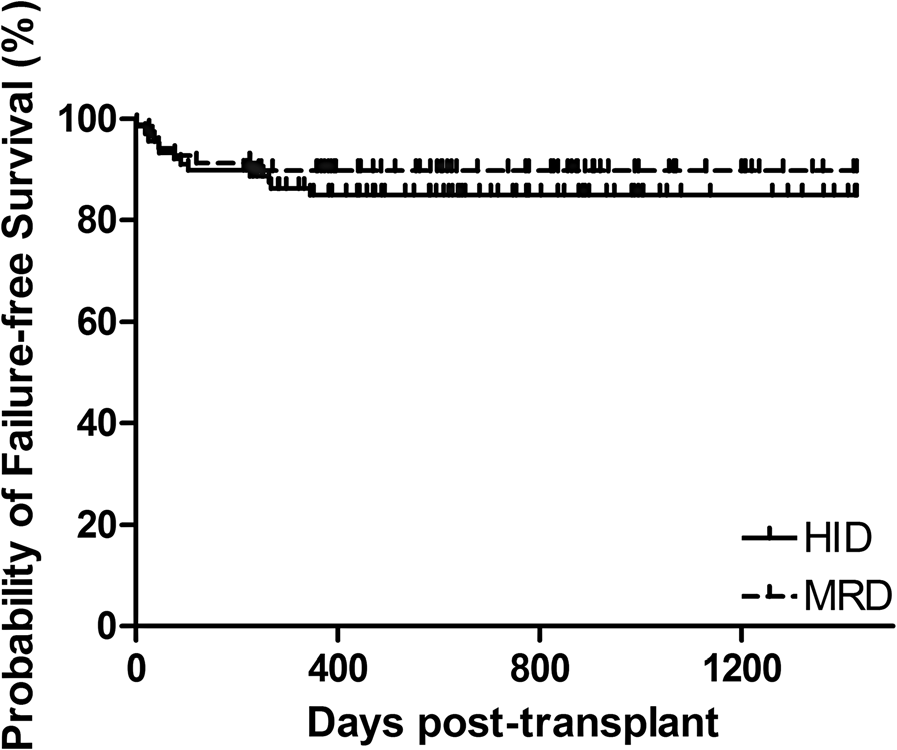
Failure-free survival of two cohorts: HID, 3-year FFS of 85.0% ± 3.9%; MRD, 3-year FFS of 89.8% ± 3.7% (*P* = 0.413)

**Fig. 4:**
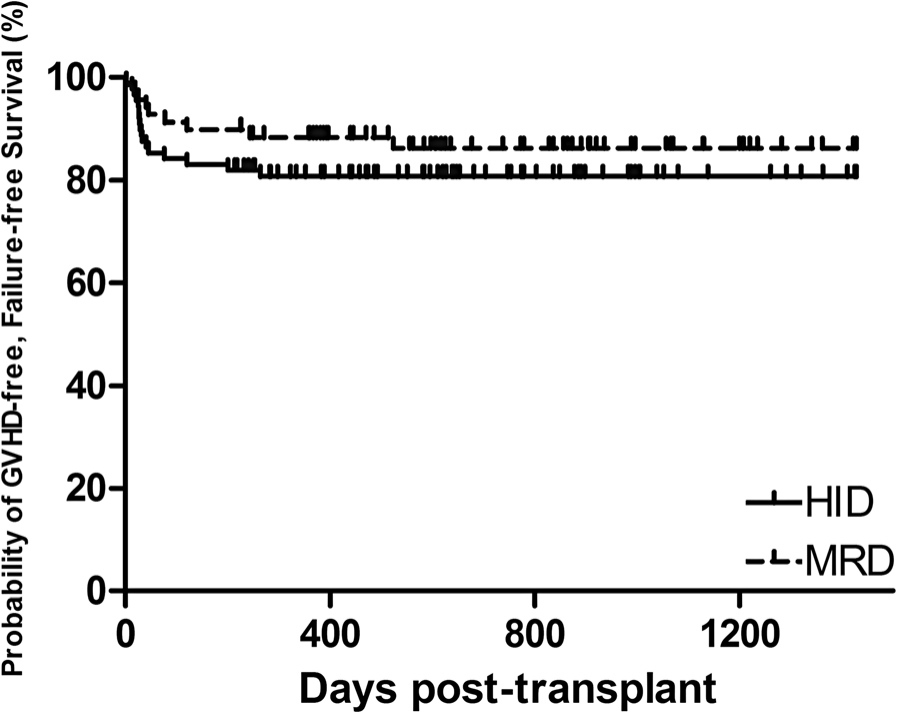
GVHD, failure-free survival of two cohorts: HID, 1-year GFFS of 80.8% ± 4.2%; MRD 1-year GFFS of 88.4% ± 3.9% (*P* = 0.282)

The three-year probabilities of overall survival (OS) were 86.1 ± 3.7% and 91.3 ± 3.4% after HID and MRD-related donor transplants, respectively (*P* = 0.358, Fig. 2). The three-year FFS was also not significantly different in the upfront HID HSCT cohort (85.0 ± 3.9%) vs. the MRD controls (89.8 ± 3.7%) (*P* = 0.413, Fig. 3). Increased RBC transfusions, longer SAA courses, and poorer performance scores significantly predicted survival outcomes in univariate analysis (Additional file 1: Table S1). In multivariate analysis, the risks of mortality did not differ significantly by donor type (Table 2), but mortality was significantly higher in patients receiving increased RBC transfusions and in those with poor performance scores. The estimated GFFS at 1 year was also similar (80.8 ± 4.2% and 88.4 ± 3.9%, *P* = 0.282, Fig. 4) in mismatched and matched patients.

As of April 30, 2016, all of the 140 surviving patients (77 HID and 63 MRD patients) achieved transfusion independence. Among the 120 patients (63 HID and 57 MRD) who were followed up for more than 1 year, 100% alive patients break away from transfusion. 87.9 vs. 88.2% had normal WBC count, 90.9 vs. 88.2% had normal platelet (PLT) count, and 93.9 vs. 91.2% cases attained hemoglobin above 100 g/L in the HID and MRD groups, respectively. In addition, 86.8% of HID and 86.7% of MRD patients achieved Karnofsky Performance Status Scale (KPS) scores ≥90.

## Discussion

Allo-HSCT from MRD leads to long-term survival in over 80% of patients, with most survivors having a normal performance status [24–27]. In general, HSCT with sustained engraftment restores bone marrow function and precludes the late complications such as relapse and clonal evolution observed with IST. More than two thirds of patients lack an MRD; however, a HID is a readily available for nearly all patients. Indeed, transplantation from HID has benefited from the same improvements as has transplantation from matched siblings, and great progress has been made in both engraftment and survival in HID [28]. Furthermore, rabbit ATG is markedly inferior to horse ATG as a first-line treatment for SAA [8]. Thus, both the unavailability of horse ATG and poor economic status which afforded either IST or SCT have prompted interest in the feasibility of upfront HID HSCT in developing countries such as China. Hence, we conducted a study based on registry data regarding the feasibility of upfront HID SCT. To the best of our knowledge, we are the first to compare outcomes of consecutive patients undergoing upfront HID SCT with upfront allo-HSCT using MRD. Data from our study demonstrated that comparable engraftment and survival outcomes were achieved in both cohorts.

Graft failure is a central problem in HSCT for SAA, occurring more frequently than in other hematological malignancies. In the initial attempts with MRD-SCT, using CY alone as conditioning regimen and MTX alone for GVHD prophylaxis, the rates of GF with MRD-SCT were over 30% [29]. Higher GF rates of 70% were also observed in early experience with HID SCT [30]. However, the current situation is considerably different. CY with ATG for conditioning followed by post-grafting CsA plus MTX resulted in an engraftment rate of 95% in matched siblings [24]. Similarly, several studies on HID transplants reported incidence of GF ranging from 0 to 25% due to recent advances in ex vivo depletion of T cells or unmanipulated in vivo regulation [21, 28, 31–35]. Our findings showed that the HID cohort without in vitro T cell depletion had myeloid (97.75 vs. 97.10%) and platelet (96.63 vs. 95.65%) engraftment rates comparable to those of the MRD cohort. As analyzed in our previous research [21], the reasons for these encouraging engraftment outcomes in mismatched transplants may be multifactorial, including adding BU to CY + ATG for intensified conditioning [36, 37], using G-CSF-mobilized grafts [38] and combining CsA, MTX, and MMF as GVHD prophylaxis.

As SAA is a non-neoplastic hematologic disorder, another goal of transplantation is to avoid acute or chronic GVHD after successful engraftment. Although higher proportions of patients with II–IV (30.34 vs. 1.45%) and III–IV aGVHD (10.11 vs. 1.45%) in the HID than in the MRD cohorts, the incidences of aGVHD were comparable to those of recent studies involving ex vivo T cell depletion haploidentical transplants, reported II–IV aGVHD rates of 30–33% [11, 12, 28, 31]. Unexpectedly, univariate analysis showed that older age (≥18 years) and PB graft were associated with a reduced incidence of II–IV aGVHD in our total cohort. However, it is important to realize that adult and PB graft were significantly more common in the matched group, and no significant factors were identified in multivariate analysis. Although we found that risks of severe aGVHD differed significantly by donor type, the incidence of severe aGVHD-related TRM was not obviously higher in the HID cohort. PTCY or CD25mAb may reduce the incidence of severe GVHD, which should be assessed in future studies.

Extensive cGVHD has a major impact on quality of life in HSCT recipients [39]. The incidence of extensive cGVHD was similar between the HID and MRD HSCT cohorts in our study. However, limited GVHD may also affect quality of life post-transplantation. We assessed quality of life during the follow-up period; most patients with limited cGVHD rated their quality of life as excellent and their symptoms as minimal or mild. Our study demonstrated that nearly 90% of patients in both cohorts who survived for more than a year achieved KFS scores above 90. Further long-term follow-up is necessary to confirm this positive performance status. Besides, similar 1-year GFFS in two cohorts also supported that patients alive had comparable survival rates without ongoing morbidity.

Infection was also a major barrier to the wider application of HID SCT. However, delayed immune reconstitution within 6 months post-transplantation was observed in our HID cohort. The possible reasons were as follows: first, an intensified immunological suppression conditioning regimen was used in the mismatched patient group to promote engraftment and prevent GVHD; second, additional immunological suppression therapy was administered because of higher incidences of aGVHD in the HID cohort. Encouragingly, the incidence of viral infection was similar between the two groups. In the HID and MRD cohorts, CMV reactivation occurred in 51.7 and 43.5% of patients, but only one mismatched patient developed disease. EBV viremia was detected in 28.1 and 21.7% of patients in each group and lymphoproliferative disorder in 1.1 and 1.4%, respectively. Ganciclovir was commonly administered as prophylaxis, intensive surveillance was performed twice weekly, and preemptive treatment was administered promptly once viremia was detected, which may have helped to decrease the incidence of lethal virus infection.

We have observed comparable survival (three-year OS of 86.1 vs. 91.3%, FFS of 85.0 vs. 89.8%) after HID and MRD transplantations, with increased RBC transfusions and poor ECOG being the adverse factors. Consistent with other reports [22, 40], heavily transfused patients have worse outcomes, including increased graft failure and poor survival. In our data, increased transfusions had no influence on either engraftment or GVHD. One possible explanation is that iron deposition brought by transfusions decreased organ function prior to SCT. Passweg et al. also observed that poor performance scores affected outcomes after transplantation [41]. All of these findings point to the need for reducing such delays pre-transplantation, which may reduce the need for multiple transfusions and the incidence of pre-treatment infections, thus improving performance status at transplantation and improving OS rates.

Our study has several limitations, including the potential for selection bias; the fact that the patients in the HID cohort were younger than those in the MRD cohort and that the donor-recipient sex match and graft source differed significantly between groups implied that comparisons between GVHD rates in these groups were not feasible. As age has been shown to influence outcomes [42], we also stratified our population into pediatric and adult patients and observed similar outcomes in pediatric HID vs. MRD and adult HID vs. MRD. Despite these limitations, our data offer compelling comparative evidence of the value of HID SCT as a front-line treatment in developing countries.

HID SCT has several obvious advantages compared to other alternative donors. First, donors are available for nearly all patients; second, SCT can be performed immediately, which is particularly crucial for patients with vSAA who need prompt therapy; and third, continued donor access is available for rescuing graft failure or for treating infections.

## Conclusions

In summary, our findings show that upfront HID HSCT is a safe and feasible choice for patients without suitably matched donors. Our data indicates that there might be a change in the SAA treatment algorithm in developing countries, and upfront HID HSCT might be considered alongside IST for patients lacking matched donors in specialized centers. Further prospective multicenter research is needed to confirm that.

## Additional file

Additional file 1: Table S1. Univariate analysis of factors associated with survival outcomes. **Table S2.** Univariate analysis of factors associated with II–IV aGVHD and III–IV aGVHD. **Figure S1a.** The cumulative incidence of 28-day neutrophil engraftment:HID97.75 ± 0.03%, MRD97.10 ± 0.05% (*P* = 0.528). **Figure S1b.** The cumulative incidence of platelet engraftment: HID96.63 ± 0.05%, MRD 95.65 ± 0.08% (*P* = 0.989). **Figure S2a.** The cumulative incidence of II–IV aGVHD: HID 30.34 ± 0.24%, MRD1.45 ± 0.02% (*P* < 0.001). **Figure S2b.** The cumulative incidence of III–IV aGVHD: HID10.11 ± 0.10%, MRD1.45 ± 0.02% (*P* = 0.026). **Figure S3a.** The cumulative incidence of cGVHD (*P* < 0.001). **Figure S3b.** The cumulative incidence of extensive cGVHD (*P* = 0.426). (DOCX 42 kb)

## Abbreviations

AA: Aplastic anemia
aGVHD: Acute graft-versus-host disease
ATG: Antithymocyte globulin
BM: Bone marrow
BU: Busulfan
CBMTR: Chinese Bone Marrow Transplantation Registry
CD25 mAb: CD25 monoclonal antibody
cGVHD: Chronic graft-versus-host disease
CMV: Cytomegalovirus
CMV-CTL: Cytomegalovirus-specific cytotoxic T lymphocytes
CsA: Cyclosporin A
CY: Cyclophosphamide
EBV: Epstein-Barr virus
ECOG: Eastern Cooperative Oncology Group
FDC: Full donor chimerism
FFS: Failure-free survival
Flu: Fludarabine
GFFS: GVHD-free, failure-free survival
HID: Haploidentical donor
HSCT: Hematopoietic stem cell transplantation
IST: Immunosuppressive therapy
KPS: Karnofsky Performance Status Scale
MMF: Mycophenolate mofetil
MNC: Mononuclear cell
MRD: Matched related donor
MTX: Methotrexate
OS: Overall survival
PB: Peripheral blood
PTLD: Post-transplant lymphoproliferative disorders
RBC: Red blood cell
RRT: Regimen-related toxicity
SAA: Severe aplastic anemia
TRM: Transplantation-related mortality
