# Supplementary material for: Upfront haploidentical transplant for acquired severe aplastic anemia: registry-based comparison with matched related transplant
Source: J Hematol Oncol. 2017 Jan 21;10:25. doi: 10.1186/s13045-017-0398-y (PMC5251320; doi:10.1186/s13045-017-0398-y)
Supplement: Additional file 1: Table S1. — Univariate analysis of factors associated with survival outcomes. Table S2. Univariate analysis of factors associated with II–IV aGVHD and III–IV aGVHD. Figure S1a. The cumulative incidence of 28-day neutrophil engraftment:HID97.75 ± 0.03%, MRD97.10 ± 0.05% (P = 0.528). Figure S1b. The cumulative incidence of platelet engraftment: HID96.63 ± 0.05%, MRD 95.65 ± 0.08% (P = 0.989). Figure S2a. The cumulative incidence of II–IV aGVHD: HID 30.34 ± 0.24%, MRD1.45 ± 0.02% (P < 0.001). Figure S2b. The cumulative incidence of III–IV aGVHD: HID10.11 ± 0.10%, MRD1.45 ± 0.02% (P = 0.026). Figure S3a. The cumulative incidence of cGVHD (P < 0.001). Figure S3b. The cumulative incidence of extensive cGVHD (P = 0.426). (DOCX 42 kb) [file 13045_2017_398_MOESM1_ESM.docx]

**Additional file**

**Table S1 Univariate analysis of factors associated with survival outcomes.**

|  |  | Probability of survival (SE) | | | | | |
| --- | --- | --- | --- | --- | --- | --- | --- |
| variable | No. | 3-year OS rate | *P* | | 3-year FFS rate | | *P* |
| Total  Donor type  HID  MRD | 89  69 | 86.1(3.7)  91.3(3.4) | 0.358 | | 85.0(3.9)  89.8(3.7) | | 0.413 |
| Sex  Male  Female | 96  62 | 88.4(3.3)  88.6(4.1) | 0.917 | | 87.3(3.4)  86.9(4.3) | | 0.996 |
| Age  Children  Adult | 40  118 | 90.0(4.7)  87.9(3.0) | 0.770 | | 90.0(4.7)  86.2(3.2) | | 0.576 |
| Previous transfusion  (RBC)  ＜10u  ≥ 10 u | 90  58 | 96.5(2.0)  77.4(5.5) | **0.000** | | 95.4(2.2)  75.7(5.7) | | **0.000** |
| SAA course (m)  ＜2  ≥2 | 87  71 | 94.1(2.6)  81.4(4.7) | **0.011** | | 91.8(3.0)  81.4(4.7) | | **0.045** |
| ECOG  0-1  2 | 117  41 | 92.8(2.5)  75.6(6.7) | **0.001** | | 92.0(2.6)  73.2(6.9) | | **0.001** |
| Graft type  BM+PB  BM  PB  MNC (×10^8/L) | 121  12  24 | 86.6(3.1)  82.5(11.3)  100.0 | 0.176  0.346 | | | 85.8(3.2)  74.1(12.9)  100.0 | 0.093  0.690 |
| ≤10 | 75 | 86.4(4.0) |  | | | 86.4(4.0) |  |
| ＞10 | 80 | 91.1(3.2) |  | | | 88.6(3.6) |  |
| CD34 (×10^6/L) |  |  | 0.714 | | |  | 0.397 |
| ≤4 | 80 | 89.9(3.4) |  | | | 89.9(3.4) |  |
| ＞4 | 75 | 87.6(3.9) |  | | | 85.0(4.2) |  |
| Donor sex  Male  Female  Donor age (y)  ＜40  ≥40  ABO match  Same/Minor mismatch | 91  67  84  73  124 | 88.7(3.4)  87.9(4.0)  90.4(3.2)  86.0(4.1)  88.6(2.9) | | 0.832  0.421  0.981 | | 86.5(3.6)  87.9(4.0)  89.2(3.4)  84.6(4.3)    87.0(3.0) | 0.829  0.428  0.807 |
| Major mismatch/Different | 34 | 88.0(5.6) |  | | 88.0(5.6) | |  |

**Table S2 Univariate analysis of factors associated with II-IV aGVHD and III-IV aGVHD**

|  |  | Probability of aGVHD(SE) | | | | |
| --- | --- | --- | --- | --- | --- | --- |
| variable | No. | II-IV aGVHD | *P* | | III-IV aGVHD | *P* |
| Total  Donor type  HID  MRD | 89  69 | 30.34(0.24)  1.45(0.02) | **＜0.001** | | 10.11(0.10)  1.45(0.02) | **0.026** |
| Sex  Male  Female | 96  62 | 16.67(0.15)  19.35(0.26) | 0.710 | 5.21(0.05)  8.06(0.12) | | 0.468 |
| Age  Children  Adult | 40  118 | 32.50(0.57)  12.71(0.09) | **0.030** | 12.50(0.28)  4.24(0.03) | | **0.058** |
| Previous transfusion  (RBC)  ＜10u  ≥ 10 u | 90  58 | 18.89(0.17)  17.24(0.25) | 0.844 | 4.44(0.05)  8.62(0.14) | | 0.295 |
| SAA course (m)  ＜2  ≥2 | 87  71 | 12.64(0.13)  23.94(0.26) | **0.062** | 3.45(0.04)  9.86(0.13) | | **0.099** |
| ECOG  0-1  2 | 117  41 | 15.38(0.11)  24.39(0.46) | 0.206 | 3.42(0.03)  14.63(0.31) | | **0.012** |
| Graft type  BM+PB  BM  PB  MNC (×10^8/L) | 121  12  24 | 22.31(0.14)  8.33(0.69)  0 | **0.023**  0.894 | 7.44(0.06)  8.33(0.69)  0 | | 0.382  0.577 |
| ≤10 | 75 | 18.67(0.21) |  | | 5.33(0.07) |  |
| ＞10 | 80 | 17.50(0.18) |  | | 7.50(0.09) |  |
| CD34 (×10^6/L) |  |  | 0.471 | |  | 0.458 |
| ≤4 | 80 | 20.00(0.20) |  | | 5.00(0.06) |  |
| ＞4 | 75 | 16.00(0.18) |  | | 8.00(0.10) |  |
| Donor sex  Male  Female  Donor age (y)  ＜40  ≥40  ABO match  Same/Minor mismatch | 91  67  84  73  124 | 18.68(0.17)  16.42(0.21)  20.24(0.19)  15.07(0.18)  18.55(0.12) | 0.767  0.346  0.582 | | 6.59(0.07)  5.97(0.09)  5.95(0.07)  6.85(0.09)  6.45(0.05) | 0.902  0.829  0.901 |
| Major mismatch/Different | 34 | 14.71(0.38) |  | 5.88(0.17) | |  |

Figure S1a. The cumulative incidence of 28-day neutrophil engraftment：HID97.75±0.03%, MRD97.10±0.05% (*P*=0.528)

Figure S1b. The cumulative incidence of platelet engraftment：HID96.63±0.05%, MRD 95.65±0.08% (*P*=0.989)

Figure S2a. The cumulative incidence of II-IV aGVHD：HID30.34±0.24%, MRD1.45±0.02% (*P*＜0.001)

Figure S2b. The cumulative incidence of III-IV aGVHD：HID10.11±0.10%, MRD1.45±0.02% (*P*=0.026)

Figure S3a. The cumulative incidence of cGVHD (*P*＜0.001)：

1-year HID30.58±0.26%, MRD4.35±0.06%

3-year HID39.30±0.54%, MRD8.35±0.13%

Figure S3b. The cumulative incidence of extensive cGVHD (*P*=0.426)：

1-year HID3.42±0.04%, MRD 0%

3-year HID3.42±0.04%, MRD 2.03±0.04%
